# Data linkage studies of primary care utilisation after release from prison: a scoping review

**DOI:** 10.1186/s12875-024-02527-w

**Published:** 2024-08-07

**Authors:** Janine A. Cooper, Siobhán Murphy, Richard Kirk, Dermot O’Reilly, Michael Donnelly

**Affiliations:** 1 Centre for Public Health, Queen’s University Belfast, Royal Hospitals Site, Grosvenor Road, Belfast, UK; 2 Administrative Data Research Centre Northern Ireland (ADRC NI), Centre for Public Health, Queen’s University Belfast, Royal Hospitals Site, Grosvenor Road, Belfast, UK; 3 South Eastern Health and Social Care Trust, Ulster Hospital, Dundonald, UK

**Keywords:** Data linkage, Primary care, Prison, Public health, Scoping review

## Abstract

**Background:**

Primary care plays a central role in most, if not all, health care systems including the care of vulnerable populations such as people who have been incarcerated. Studies linking incarceration records to health care data can improve understanding about health care access following release from prison. This review maps evidence from data-linkage studies about primary care use after prison release.

**Methods:**

The framework by Arksey and O’Malley and guidance by the Joanna Briggs Institute (JBI) were used in this review. This scoping review followed methods published in a study protocol. Searches were performed (January 2012-March 2023) in MEDLINE, EMBASE and Web of Science Core Collection using key-terms relating to two areas: (i) people who have been incarcerated and (ii) primary care. Using eligibility criteria, two authors independently screened publication titles and abstracts (step 1), and subsequently, screened full text publications (step 2). Discrepancies were resolved with a third author. Two authors independently charted data from included publications. Findings were mapped by methodology, key findings and gaps in research.

**Results:**

The database searches generated 1,050 publications which were screened by title and abstract. Following this, publications were fully screened (*n* = 63 reviewer 1 and *n* = 87 reviewer 2), leading to the inclusion of 17 publications. Among the included studies, primary care use after prison release was variable. Early contact with primary care services after prison release (e.g. first month) was positively associated with an increased health service use, but an investigation found that a large proportion of individuals did not access primary care during the first month. The quality of care was found to be largely inadequate (measured continuity of care) for moderate multimorbidity. There were lower levels of colorectal and breast cancer screening among people released from custody. The review identified studies of enhanced primary care programmes for individuals following release from prison, with studies reporting a reduction in reincarceration and criminal justice system costs.

**Conclusions:**

This review has suggested mixed evidence regarding primary care use after prison release and has highlighted challenges and areas of suboptimal care. Further research has been discussed in relation to the scoping review findings.

**Supplementary Information:**

The online version contains supplementary material available at 10.1186/s12875-024-02527-w.

## Background

More than 10.7 million people are incarcerated globally [1]. The prison population is recognised by the United Nations as a vulnerable and marginalised group that may be subject to discrimination and exclusion after release [2]. Health care in prison differs between countries, and after release from incarceration, individuals will navigate different health services and systems [3–5]. Policies for health care after release from prison (including the transfer or sharing of medical information) may improve the transition to primary care, and continuity of care may expedite linkage with appropriate community services for individuals with low engagement during a time of increased vulnerability [5]. Co-ordinating re-entry into society after release from prison can be difficult, for example individuals may experience limitations in accessing health and social services, housing and employment [6]. Furthermore, there is an elevated risk of morbidity and mortality after release from incarceration [7–10].

Studies linking incarceration records to health care data can help identify trends and/or patterns in use of different health care settings following release from prison, determine facilitators/difficulties in accessing care and health-related or other outcomes, which can help profile people who are most at risk following release from prison. Research on emergency department attendance after release from prison and reason(s) for attending is important to help understand who is most likely to require urgent care and when. For example, a cohort study in Canada, reported higher rates of emergency department use during the two years after release compared to the general population, with rates highest in the week after release from prison [11]. Most emergency department visits in the 2 years post-release were classed as high urgency (and commonly related to injury and mental health disorders) [11].

This review focuses on primary care use after release from prison due to reports about access problems, low uptake and poor connectivity. For example, a retrospective cohort study in Canada linked prison correctional services data and health administrative data and reported a significantly lower primary care attachment (i.e. use of a community health centre, enrolment in a primary care model or history of primary care fee codes) among individuals during the two years before entry into prison and in the two years after release (in comparison to the general population) [12]. During the two years after release, approximately one-quarter of people with specific chronic conditions were not attached to primary care [12]. Using the same linked data cohort in Canada, a separate retrospective study reported that approximately two-thirds of women and three-quarters of men did not access primary care during the first month after release from prison [13]. However, the study reported a higher relative rate of primary care use among people in prison and post-release compared to the general population [13].

## Purpose of this scoping review

The evidence around data-linkage studies of primary care use after release from prison has not been mapped and this review aims to address this gap. This review will be used to inform research undertaken by our ESRC-funded Administrative Data Research Centre (ADRC) about health after release from prison and therefore will focus on observational record-linkage studies in keeping with the remit of ADR UK. For example, the use administrative data and the ability to draw from multiple data sources can accurately assess health outcomes after prison release at various time points (i.e. first month or year) thereby maximising potential for timely intervention and post-release follow-up for at-risk individuals. In this review, we will scope the research literature on record linkage studies about primary care after prison release to identify, map and summarise studies, and will report, compare and comment on methodologies used to conduct this research, for example, study designs, outcomes and gaps in knowledge. This review may be used to inform future epidemiological research studies and targeted interventions for people at-risk following release from prison, leading to improvements in continuity of care.

## Methods

Detailed methods for this scoping review have been published in a study protocol [14]. Five stages of the framework by Arksey and O’Malley were used in this review, and the Joanna Briggs Institute (JBI) guidance was consulted during the development stage [15, 16]. A completed Preferred Reporting Items for Systematic reviews and Meta-Analyses extension for Scoping Reviews (PRISMA-ScR) checklist is provided in appendix 1) [17].

### Stage 1: identifying the research questions

As published in the study protocol [14], the scoping review questions were:

1. What is the scope of the research literature on record linkage studies about primary care after prison release?
2. What methodologies are reported in these studies?
3. What are the findings in relation to primary care contact by people released from prison (including any hand-over arrangements and accessing and using primary care) and any reported health or prison related outcomes?
4. Where are the knowledge gaps in this area?

### Stage 2: identifying relevant studies

A MEDLINE search strategy relating to (i) people who have been incarcerated and (ii) primary care was developed by JAC and MD, and refined by the Subject Librarian for the School of Medicine, Dentistry and Biomedical Sciences in Queen’s University Belfast (published with the study protocol [14]). Separate search strategies for EMBASE and Web of Science Core Collection were developed by JAC and MD. All search strategies used in this scoping review are provided in appendix 2. The literature databases were searched from January 2012 to March 2023 to review the most recent literature. The search strategies included publications available in English only (due to resources for translation). Grey literature was not searched as part of this review. References of included studies were screened by JAC to identify any additional publications.

### Stage 3: study selection

MEDLINE, EMBASE and Web of Science Core Collection searches were performed by JAC on 29th March 2023 with the results combined in Endnote (Reference Manager) and duplicate publications removed. The inclusion and exclusion criteria used to determine study eligibility (as per study protocol [14]) are provided in Table 1. Two authors (JAC and SM) independently screened titles and abstracts (step 1). Publications were subsequently screened in full (step 2), if the publication seemingly met the eligibility criteria in step 1, or if there was any uncertainty regarding eligibility. Two authors (JAC and SM) independently screened full publications in step 2, and any disagreements in eligibility were discussed with a third author (MD).

**Table 1:** Inclusion and exclusion criteria used to determine study eligibility (as per study protocol [14])

**Population**
Studies among adults released from prison into the community will be eligible for inclusion. There will be no exclusions on time periods after release from prison, but where possible this will be recorded in the data charting form
**Concept**
Studies addressing any contact with primary care health services after release from prison will be eligible for inclusion. As part of this selection process, the term ‘primary care contact’ will involve all types of health services provided within the general practice setting (for example, in-person and telephone consultations, home visits, clinic and treatment appointments). This will also include similar terminology used for these services, such family health, family physician, primary care physician and nurse practitioner etc. Contact with other primary care services such as a community pharmacy, or dental and optometry services will also be eligible for inclusion. All observational studies (i.e. cohort, case-control and cross-sectional studies) using linked data from two or more sources will be eligible for inclusion
**Context**
All geographical locations will be eligible for inclusion. Only research from peer-reviewed journal articles will be included. All sources of data other than peer-reviewed academic journal papers, for example, conference abstracts, editorials, commentaries and letters will be excluded

### Stage 4: charting the data

In the protocol development, a data charting form was piloted by two authors [14]. Before conducting stage 4 of the review, the charting form was retested. Two authors (JAC and SM) extracted information from a sample of the included studies (*n* = 3) using the charting form and discussed the consistency and accuracy of the recorded data. These discussions resulted in further modifications to the charting form. The data charting form used in the review is provided in appendix 3. The two authors (JAC and SM) who tested the form, subsequently charted data from included publications independently. Any discrepancies in the charting forms were initially discussed by the two extracting authors (JC and SM); and any resulting disagreements were discussed with a third author (MD). Some corresponding authors of included publications were contacted for further information at this stage.

### Stage 5: collating, summarising and reporting the results

**Fig. 1:**
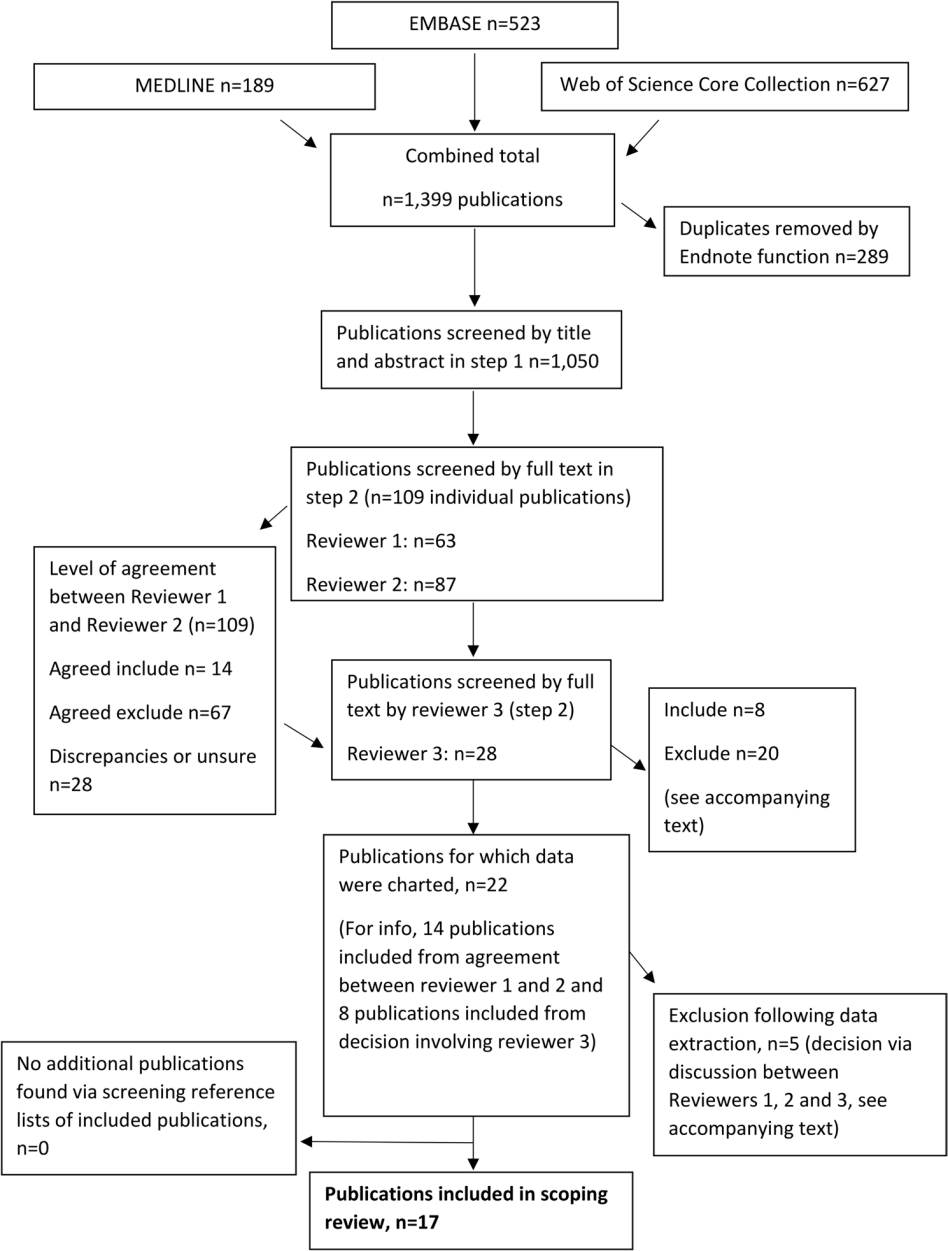
Flow diagram showing the review process

A flow diagram of the review process (including search results and study selection stages) is shown in Fig. 1. Information collated in the charting forms was entered into Microsoft Excel sheets for data management and cleaning. To answer the review questions, findings were mapped by methodology, key findings and gaps in research and summary tables (for example, key characteristics and methodological features of included studies) were populated.

### Ethics approval and stakeholder engagement

Ethical approval was not required (as the review comprised publicly available sources of research). The review was discussed regularly at meetings with staff representatives from the Northern Ireland Healthcare in Prison Service (HIPS) including the Clinical Director and senior management of HIPS.

## Results

A total of 1,339 publications (MEDLINE *n* = 189, EMBASE *n* = 523 and Web of Science Core Collection *n* = 627) were identified. Following the removal of 289 duplicate publications, 1,050 publications were screened by title and abstract (step 1). 63 publications were fully screened by reviewer 1. 87 publications were fully screened by reviewer 2. Following full screening, 28 publications were discussed with reviewer 3 (as a result of discrepancies between reviewer 1 and 2). Of the 28 publications screened by reviewer 3, 13 were excluded, 2 were included and the remaining 13 were discussed with reviewer 1 (as there was no agreement or an unsure decision remained). Of the 13 publications discussed, six were included and the remaining seven publications were excluded (reasons for exclusion were no data linkage [18–23] and no substantial data on primary care use [24]). Therefore, data were charted for 22 publications. However, five studies passed through the earlier screening stages but on closer inspection of the full text at data extraction stage, the paper(s) did not contain data about the key variables and outcomes of interest. The reasons for exclusion at this stage were: no results on health service use data [25], unclear data regarding where and when there were opportunities for this population to avail of primary care-based screening [26], medical care in study not restricted to primary care [27], primary care visits may have occurred before or after a participant’s involvement in correctional control [28], health data not restricted to primary care [29]. In total, 17 publications were included in the review. Reference lists of the 17 included studies were screened by one author (JAC); no additional publications were added to the review.

**Table 2:** Characteristics of included studies

Author	Year of publication	Journal name	Custodial setting	Primary care service(s)	Setting location (country)	Primary and secondary outcomes	Quality assessment
Calais‑Ferreira [30]	2022	BMC Health Services Research	Prison	After release: Medicare (universal health insurance scheme). Primary care encounters included services provided by primary care doctors and nurse practitioners	Australia	Quality of primary healthcare measures: continuity of care and use of extended consultations. Outcomes: Usual Provider Continuity Index (UPCI), Continuity of Care (COC) Index and at least one extended primary care consultation (> 20 min)	RECORD
Carroll [31]	2017	Medical Journal of Australia	Prison	Primary care attendance (defined by Medicare item descriptions). GP attendance included services directly provided by GPs, practice nurses or Indigenous health workers on behalf of a GP	Australia	Rates of GP attendance during the 2 years after prison release	Not stated
Dirkzwager [32]	2021	The Lancet Regional Health - Europe	Pre-trial detention centres and prisons	GP recorded information	The Netherlands	Health problems 1 year pre-/post-prison. Health problems (dichotomous) i.e. ‘attending a GP for a specific health problem ≥ 1 times that year’, or ‘not presenting that health problem or did not visit their GP at all in that year’	Not stated
Harvey [33]	2022	BMC Health Services Research	Prison	Transitions Clinic Network (TCN) programme existing community health centre and enhanced primary care to people released from correctional facilities who have a chronic health condition or > 50 years. Medicaid claims data: calculate year cost for each participant	USA	Not clear Costs associated: TCN program; Medicaid/criminal justice system	Not stated
Howell [34]	2016	Journal of General Internal Medicine	Jail, prison, detention center, or juvenile correctional facility	Primary care engagement: defined as ≥ 2 primary care visits, at least 90 days apart in the period 12 to 24 months prior to the survey and, separately, the immediate 12 months after the survey	Not stated	Measured blood pressure control (clinical data in the year after the survey). Primary care engagement. Measured receipt of antihypertensive medications in the year after the survey (classification codes in pharmacy refill data), and calculated the medication possession ratio (percentage of days with antihypertensive medication)	Not stated
Khanna [35]	2019	AIDS Care	Provincial prison (representing all provincial facilities, including jails, detention centers, and correctional centers)	Primary care use. Ambulatory care defined as primary or specialty care, but not emergency department care	Canada	Rates of primary care use	Not stated
Kouyoumdjian [13]	2018	BMC Health Services Research	Provincial prison (representing all provincial correctional facilities, including jails and detention centres)	Visits to general practitioners or Family Physicians (walk-in clinics/community practices)	Canada	Primary care use	None stated
Kouyoumdjian [12]	2019	Canadian Family Physician	Provincial prison	Any defined use of primary care as primary care attachment	Canada	Primary care attachment and team-based primary care attachment. Baseline: 2 years before incarceration; follow-up: 2 years after release	STROBE and RECORD
Kouyoumdjian [36]	2020	PLoS ONE	Provincial prison (representing all provincial correctional facilities, including jails, detention centres, and correctional centres)	Access to care after hospital discharge; Ontario Health Insurance Plan data on primary care use	Canada	30-day medical-surgical readmission to hospital. Examined access to care after hospital discharge including primary care	STROBE
Mahentharan [37]	2021	The Canadian Journal of Psychiatry	Provincial correctional facilities (remand and sentences < 2 years)	Health service use which included ‘primary care physician visits’	Canada	Primary outcome: time to reincarceration. Secondary outcomes: correctional events and health service use. Primary care (time to first contact after release): primary care physician contact (all, mental health–related and non-mental health–related contact)	None stated
McConnon [38]	2019	American Journal of Preventive Medicine	Provincial facilities, including jails, detention centers, and correctional centers	Visits to general practitioners or family physicians. Primary care use in the 3 years before and after the index date	Canada	Primary outcomes: individuals (screen-eligible) overdue for breast/colorectal cancer screening on index date	STROBE and RECORD
Norris [39]	2021	Journal Of Women’s Health	Provincial prisons	Primary care visits	Canada	Primary care visits	None stated
Palis [40]	2022	JAMA Network Open	Provincial correctional centers	Mental health services access (including primary care)	Canada	Release to reincarceration, with/without access to mental health services. Influence of mental health services access on time to reincarceration	STROBE
Palis [41]	2022	Substance Abuse Treatment, Prevention, and Policy	Provincial prisons	Community Opioid agonist treatment (OAT) dispensation within two days of release from incarceration	Canada	Community OAT dispensation within two days of release	None stated
Wang [42]	2012	American Journal of Public Health	Recruitment - state prisons. The Jail Health Services database - County Jail	Arms: (1) Transitions Clinic: primary care team - primary care provider (experience with formerly incarcerated patients) and trained/certified community health worker (CHW) (personal history of incarceration) or (2) expedited primary care appointment at another safety-net clinic	USA	≥ 2 visits to the study-assigned primary care clinic	None stated
Wang [43]	2019	BMJ Open	Prison	Transitions Clinic Network: primary care centres (care for people recently released from incarceration)	USA	Primary outcome: reincarceration within 12 months. Secondary outcomes: preventable emergency department visits, hospitalisations and length of hospital stays	None stated
Young [44]	2015	BMJ Open	Prison	Self-reported use of mental health, alcohol and other drug, hospital, and subsequent primary care physician services in the community	Australia	Rates for hospital, mental health, alcohol and other drug and subsequent primary care physician service use Self-reported Primary Care Physican (PCP) service use within 1 month of release. Outcomes were self-reported use of mental health, alcohol and other drug, hospital, and subsequent PCP services in the community [subsequent PCP service use (3 and 6 month follow-up)]	None stated

**Table 3:** Methodological features of included studies

First author, year	Study design	Method of data linkage	Years of data linked	Time period examined after prison release	Re-incarceration
Calais‑Ferreira 2022 [30]	Prospective cohort study	Not stated	August 2008 - July 2010	From prison release date for 2 years, or until death (if death within this period)	Time spent in prison during reincarceration was excluded from follow-up time
Carroll 2017 [31]	Prospective cohort study	Probabilistic linkage	July 2008 - June 2012	2 years after release	Re-incarceration dates during follow-up were provided by the Queensland Corrective Services. Rate calculated after deducting subsequent time in prison (excluded from Medicare)
Dirkzwager 2021 [32]	Matched cohort study	One-on-one linkage of health data, prison data, and socioeconomic data performed using pseudonyms	System of Social Statistical Datasets and NIVEL Primary Care Database from 2013–2016. Dutch National Prison Database from 2014–2015	1 year before and after detention	Not stated
Harvey 2022 [33]	Propensity matched study	Not stated	2013–2016	12 months	Transitions Clinic Network (TCN) participants and the comparison group were covered by Medicaid for the duration of the study period, unless participants were re-incarcerated
Howell 2016 [34]	Prospective, multi-site observational cohort study	Not stated	Survey: 1 October 2009–30 September 2010. Primary care visits in 12 months following the survey	12 months	Not stated
Khanna 2019 [35]	Retrospective cohort study	Ontario Health Insurance Plan (OHIP) number or probabilistically	Unclear	30, 90 and 365 days after release	Index date was initial release in 2010, used the subsequent release if the person had any subsequent releases in 2010. Censored the period of follow-up at any subsequent admission to provincial prison
Kouyoumdjian 2018 [13]	Retrospective cohort study	Ontario Health Insurance Plan (OHIP) number. If unavailable, linked deterministically or probabilistically using name and date of birth	2005–2015	2 years post-release	Right censored the post-release period of follow up at re-admission to provincial prison (for persons released from provincial prison)
Kouyoumdjian 2019 [12]	Retrospective cohort study	Deterministic or probabilistic linkage	2005–2015	2 years before admission to provincial prison and 2 years after release	During the 2-year follow-up period, no exclusion on basis of death or readmission to custody. For the prison release group, to examine primary care access in the community, study excluded any time in provincial prison
Kouyoumdjian 2020 [36]	Retrospective cohort study	Unique encoded identifiers	2005 and 2015	6 months after release	Not stated
Mahentharan 2021 [37]	Retrospective cohort study	Unique person identifiers / unique encoded identifiers	2005–2015	Index release (2010) to a maximum follow-up date of 31 December 2015	Primary outcome of this study was reincarceration
McConnon 2019 [38]	Retrospective cohort study	Unique encoded OHIP, or deterministic or probabilistic linkage	2005–2015	3 years	Not clear
Norris 2021 [39]	Retrospective cohort study	Not stated (cited Kouyoumdijian et al. 2018. The health care utilization. Unique identifier or deterministic/ probabilistic linkage method) [Kouyoumdijian et al. 2018]	2005–2015	730 days after the date of release	Person-time at risk did not include any subsequent incarcerations
Palis 2022 [40]	Cohort study	Personal identification number	1 January 2015–31 December 2018	End of the study period (31 December 2018) or death	Reincarceration was the outcome of interest
Palis 2022 [41]	Cohort study	Not stated	1 January 2015–29 December 2018	Two days	Exposure of interest was stimulant use disorder (StUD) diagnosis. The exposure was determined at time of release (for each release), so this was time varying and could change from a release to the next. Covariates included number of prior incarcerations (by time of release, retrospectively to January 2015). Generalized estimating equation (GEE) - odds of Opioid agonist treatment (OAT) dispensation within two days after release was adjusted for multiple releases for the same person
Wang 2012 [42]	RCT	Not stated	(Enrollment) 15 November 2007–30 June 2009	12 months	Secondary outcome was return to jail (any incarceration in the San Francisco County Jail, and time to first incarceration)
Wang 2019 [43]	Quasi-experimental study	Identifiers: deterministic /probabilistic	2013–2016	12 months	Primary outcome of interest was reincarceration
Young 2015 [44]	Prospective cohort study	Deterministic linkage (unique prison identification number). Probabilistic data linkage with Australian National Death Index	1 August 2008–31 July 2010	Primary care physician contact within 1 month of prison release Health service utilisation within 6 months of prison release	Interviews were conducted in custody for participants reincarcerated at follow-up. Analyses used a multivariate Andersen-Gill extension of a Cox proportional hazards model. The model used robust SEs for use with multiple failure time-interval data and interval-truncation (for periods of reincarceration). Reincarcerations within follow-up were truncated (i.e. interval-truncation) (participants were not ‘at-risk’ of using community health services while incarcerated)

### What is the scope of the research literature on record linkage studies about primary care after prison release?

The characteristics of the included studies are provided in Table 2. Of the 17 studies included in this review, 12 were published since 2019. The included publications were across 14 different journals; the most common journal was BMC Health Services Research (*n* = 3). The included studies were published across four different locations. The locations were Canada (*n* = 9), Australia (*n* = 3), USA (*n* = 3), The Netherlands (*n* = 1) and in one publication the location was not stated. Included publications investigated primary health care during the transition from prison to community living. The custodial settings differed between studies, for example, ‘prison’ was commonly reported, as was ‘provisional prison(s)’ e.g. representing all provincial correctional facilities, including jails, detention centres, and correctional centres. The most common health care use investigated in included studies was primary care visits (for example use, time to first contact and quality of care).

Further characteristics of the included studies are provided in Appendix 4, including sources of data. The number of participants ranged from 94 to 48,861 (Appendix 4). The largest studies were Kouyoumdjian et al. 2018 [13] and Kouyoumdjian et al. 2019 [12]. The smallest studies were Harvey et al. 2022 [33] and Wang et al. 2019 [43]. Age was reported in eight of the included studies but was not provided in five studies and was unclear in one study. Three studies reported the inclusion of ‘adults’ but did not specific a cut-off age (Appendix 4). Sex or gender was reported in all included studies (Appendix 4). Race or ethnicity was reported 14 studies (Appendix 4).

Five publications reported using a checklist or technique for quality assessment (Table 2). One study reported using Reporting of studies Conducted using Observational Routinely-collected health Data (RECORD) statement [30]. Two studies reported using the Strengthening the Reporting of Observational Studies in Epidemiology (STROBE) checklist [36, 40]. Two studies reported using both STROBE and RECORD [12, 38].

### What methodologies are reported in these studies?

The methodological features of the included studies are provided in Table 3. The study design was stated in all included publications. The most common design was a cohort study (*n* = 14). Other designs (as defined) included a propensity-matched study, a quasi-experimental study, and a randomised controlled trial (RCT). The most common method of data linkage was linkage by unique identifiers. The methods of data linkage were not reported in six of the included publications, although one study cited methods published in another study. The most common time periods examined after release from prison were one- or two-years follow-up. The years of linked data was provided in 16 publications (in one study the years were unclear). The earliest year of data linked was 2005 and the most recent was 2018.

Re-incarceration poses potential methodological issues for the study of primary care use. If a person returned to prison or had repeated incarcerations during the study follow-up period, the individual would have varied access to primary care services. For example, data would not be collected in primary care records during re-incarceration periods when individuals are in custody (rather than community). Dates of subsequent incarcerations may be used to determine when primary care could not be accessed by an individual, otherwise there may be an assumption that an individual was still living in the community setting but was not accessing primary care services. The number of repeat incarcerations over a defined study (and duration of time spent in custody), can provide insight into recidivism patterns, risk and outcomes (for example, increased risk of morbidity and mortality after release from prison). The methods used to address re-incarceration were excluding time spent in prison during re-incarceration [12, 30, 31, 39], censoring follow-up at re-incarceration [13, 35] e.g. right censoring the post-release follow up period at the earliest of death, loss of health insurance eligibility, re-admission to prison or two years post-release [13], using re-incarceration as a study outcome [37, 40, 42, 43], measuring the study exposure of interest (e.g. stimulant use disorder diagnosis) as time varying for each release from prison [41], and using interval-truncation for periods of re-incarceration(s) during the follow-up period (multivariate Andersen-Gill extension of a Cox proportional hazards model) as individuals were not ‘at-risk’ of using health services in the community setting while in custody [44]. However, some publications did not address repeated incarceration during the study period or methods were unclear [32, 34, 36, 38]. In one study, claims data were not covered during re-incarcerated [33].

### What are the findings in relation to primary care contact by people released from prison (including any hand-over arrangements and accessing and using primary care) and any reported health or prison related outcomes?

A summary of the outcomes reported in the included studies is provided in Appendix 5. Most of the included studies reported primary care use during the follow-up period (including rates of use, visits, access, time to first contact, attachment, attending a general practice for a specific health problem and quality of care). Table 4 shows the investigations using data to report the primary care use after release from prison. Other reported outcomes included community dispensed medications, breast and colorectal cancer screening, timeliness of mental health services access and health care services costs.

**Table 4:** Reported primary care use in included studies

Primary care use	Included study
Rates of use, visits, access or attachment to primary care	Carroll et al. 2017 [31]; Howell et al. 2016 [34]; Khanna et al. 2019 [35]; Kouyoumdjian et al. 2018 [13]; Kouyoumdjian et al. 2019 [12]; Kouyoumdjian et al. 2020 [36]; Norris et al. 2021 [39]; Wang et al. 2012 [42]; Wang et al. 2019 [43]; Young et al. 2015 [44]
Time to first contact	Mahentharan et al. 2021 [37]
Attending a general practice for a specific health problem	Dirkzwager et al. 2021 [32]
Quality of care	Calais‑Ferreira et al. 2022 [30]

A summary table of the reported results from the included studies is provided in Appendix 6. Findings are mapped by the following categories:

#### Primary care use

Of the 17 included studies, 13 studies investigated primary care use (including quality, attendance, engagement and attachment) after release from prison [12, 13, 30–32, 34–37, 39, 42–44].

A cohort study investigated multimorbidity and quality of primary care in the two years after release from prison [30]. The study reported that people with moderate (2–3 domains) and complex (≥ 4 domains) multimorbidity were more likely to have a high rate of primary care contact (defined as ≥ 9 contacts per-person/year) [30]. The study measured continuity of care via the Usual Provider Continuity Index (UPCI) and the index of Continuity of Care (COC) [30]. Complex multimorbidity was associated with adequate continuity of care [UPCI (AOR 1.83, 95%CI 1.20–2.80), COC (AOR = 1.87; 95%CI 1.22–2.84), and ≥ 1 long consultation (≥ 20 min) (AOR 2.52, 95%CI 1.59–4.00)] [30]. However, moderate multimorbidity was not associated with adequate continuity of care [(UPCI or COC) but was associated with ≥ 1 long consultation (AOR 1.64, 95%CI 1.14–2.39) in the two-year period after release from prison [30].

A cohort study investigated rates of GP attendance after release from prison [31]. GP attendance was higher for people after prison release than the general population (standardised rate ratios (SRR) 2.04, 95%CI 2.00-2.07) [31]. In the two-year follow-up after release, most people (87%) had ≥ 1 contact with a GP [31]. Among people released from prison, GP attendance rates were higher for people with a history of risky opiate use (Adjusted incidence rate ratios (IRRs) 2.09, 95%CI 1.65–2.65), diagnosis of a mental health condition (Adjusted IRR 1.32, 95%CI 1.14–1.53) or medication in prison (Adjusted IRR 1.82, 95%CI 1.58–2.10) [31]. However, people who have been incarcerated with a history of risky methamphetamine use had a lower GP attendance rate (Adjusted IRR 0.71, 95%CI 0.58–0.88) [31].

A cohort study examining mental and physical health conditions found complex health needs both before and after incarceration, with custody having neither a health deteriorating nor improving effect [32]. The study examined any GP contact for a specific health condition in a one-year period. The examination of health changes among males, pre- to post-incarceration, found only one statistically significant change, with males more likely to report circulatory problems in the year post-incarceration (compared to year pre-detention) (OR 1.36, 95%CI 1.04–1.79) [32]. There were no differences in the change in prevalence rates for health conditions from pre- to post-detention between males released from prison and controls [32]. Among females there was a significant difference in the changes in general and unspecified health problems (Ratio of Odds Ratios: OR 1.92, 95%CI 1.05–3.53) [32]. Among females, pre- to post-incarceration, the prevalence of general and unspecified health problems increased, but for the controls, the prevalence of such health problems decreased [32].

An observational study used survey data regarding incarceration history (as part of data-linkage) to determine the influence of primary care engagement on the relationship between incarceration history and blood pressure control [34]. People with recent incarceration were 1.5 times more likely to have uncontrolled blood pressure compared to people never incarcerated (AOR 1.57, 95% CI: 1.09–2.26; covariates adjusted for in this model included primary care engagement pre-survey) [34]. Primary care engagement post-survey was not associated with incarceration history (exposure) and uncontrolled hypertension (outcome) [34].

A cohort study examined rates of primary care use after release from prison for people with HIV [35]. Rates of primary care use among people with HIV released from prison were higher at 30, 90 and 365 days after release compared to control groups (controls were individuals released from prison HIV-negative, general population HIV-positive and general population HIV-negative) [35]. The study found that after release from prison, people with HIV experienced a longer time to first contact with HIV ambulatory care, and rates of health care use across health care settings were elevated [35].

A cohort study examined primary care use among people released from prison (compared to the general population) [13]. The study found that primary care use was high in prison and after release [13]. An investigation of the time to first contact with primary care after prison release found low access to primary care during the first month (for example, in the month after release, 66.3% of women and 75.5% of men had not used primary care; within three months after release, 50.5% of women and 62.9% of men had not used primary care, and within two years after release, 16.8% of women and 28.2% of men had not used primary care) [13].

A cohort study which examined the use of primary care in the two years prior to entering prison and two years after release found lower primary care attachment among people who have been incarcerated compared to the general population [12]. Attachment to primary care in two years after release was 63.0% in people released from prison and 84.4% in the general population corresponding period (*P* < .001) [12]. Attachment to any team-based primary care in the two years after release was 19.9% in people released from prison compared to 21.6% in the general population corresponding period (*P* < .001) [12].

A cohort study examined (1) the 30-day medical-surgical readmission to hospital for people who have been incarcerated and (2) access to care after hospital discharge including primary care [36]. Compared with the general population, people in prison were more likely to access primary care in the 7 days after hospital discharge and people recently released from prison were more likely to access emergency department care in the 30 days after hospital discharge [36].

A cohort study examined reincarceration of people with schizophrenia after release from custody with secondary outcomes investigating correctional events and health service use (including primary care) [37]. The study found that reincarceration was higher among people with schizophrenia [37]. People with schizophrenia had higher rates of all primary care health service use (i.e. primary care physician visits including all contact, and mental health–related and non-mental health–related contact) in the 5 years following release from custody compared to people without schizophrenia [37].

A cohort study compared the health of females following release from prison with two control groups: (1) males released from prison and (2) females in the general population [39]. The study found higher morbidity and specific psychiatric conditions among females who have been incarcerated compared with the control groups, and after release from prison, females had higher rates of primary care use in all periods (follow-up until 730 days post-release) compared to the controls [39].

A randomized controlled trial compared two interventions in primary care after release from prison: (1) a Transitions Clinic consisting of a primary care provider (who had experience working with people after incarceration) and a community health worker (with personal history of incarceration) and (2) an expedited primary care appointment at another clinic) [42]. The trial reported similar rates of primary care use in both arms (Transitions Clinic 37.7% vs. expedited primary care appointment 47.1%) after 12 months of follow-up [42]. However, the Transitions Clinic arm had lower rates of emergency department use compared to expedited primary care appointment (25.5% vs. 39.2%, *P* = .04) [42].

A quasi-experimental study examined the provision of enhanced primary care (Transitions Clinic Network (TCN) providing increased access to primary care services following release from prison) and criminal justice system contact [43]. The study found that people experiencing enhanced primary care after release from prison were less likely to return to prison for a parole or probation technical violation (AOR 0.38, 95%CI 0.16–0.93) and have fewer incarceration days (adjusted incidence rate ratio: 0.55, 95%CI 0.35–0.84) [43].

A cohort study investigated the relationship between primary care physician contact during the first month after prison release and health service use in the six months following release [44]. The study found that contact with primary care physician services early after prison release increased health service use [44]. Primary care physician contact in the first month after release was positively associated with hospital health services use (AHR 2.07, 95%CI 1.39–3.09), mental health services use (AHR 1.65, 95%CI 1.24–2.19), alcohol and other drug health services use (AHR 1.48, 95%CI 1.15–1.90) and further primary care physician service use (AHR 1.47, 95%CI 1.26–1.72) during a sixth month follow-up [44].

#### Mental health services access

One included study investigated mental health services access after release from prison [40]. The cohort study reported that mental health services access was associated with reduced reincarceration risk (HR 0.61, 95%CI 0.39–0.94) [40]. However, this risk was increased by each additional month post-release before access to mental health services [40]. The reincarceration risk was significantly higher for outpatient emergency care (mental health services) (HR 1.41, 95%CI 1.08–1.83) compared with outpatient primary care (mental health services) [40].

#### Community dispensed medications

Two studies examined community dispensed medications after release from prison [34, 41]. The first included study was an observational design which used mediation analyses to determine the influence of antihypertensive adherence (via pharmacy refill data) on the relationship between incarceration history and blood pressure control [34]. The study reported that recent incarceration was associated with uncontrolled blood pressure after release (in people with a history of hypertension) [34]. People with recent incarceration were 1.5 times more likely to have uncontrolled blood pressure compared to people never incarcerated (AOR 1.57, 95%CI: 1.09–2.26; covariates adjusted for in this model included primary care engagement prior to the survey) [34]. However, the adjusted odds ratio of recent incarceration compared to never incarceration was not significantly impacted with the addition of antihypertensive adherence (AOR 1.58, 95%CI: 1.09–2.27) [34]. The second included study that examined community dispensed medications after release from prison was a cohort study investigating the impact of stimulant use disorder diagnosis on post-release opioid agonist treatment dispensation [41]. The study reported that approximately one-quarter of individuals with an opioid use disorder received opioid agonist treatment dispensation within two days of release from prison [41]. Furthermore, the study found that individuals with mental illness (based on one hospital record/two outpatient records during one year for either anxiety, depression, schizophrenia, bipolar, personality or stress disorder) and stimulant use disorder were less likely to receive opioid agonist treatment post-release (AOR 0.73, 95%CI 0.64–0.84) [41].

#### Cancer screening

One included study investigated cancer screening after release from prison [38]. The cohort study reported lower levels of colorectal and breast cancer screening among people released from custody compared to the general population [38]. The study found that people released from custody were more likely to be overdue screening for colorectal (ARR 1.44, 95%CI 1.42–1.46) and breast cancer (ARR 1.99, 95%CI 1.83–2.17) compared with the general population, and more likely to still be overdue screening three years later [38].

#### Cost saving of primary care program

One included study investigated costs of a primary care program for people recently released from prison [33]. The propensity matched study evaluated the Transitions Clinic Network (TCN) programme, a comparison of existing community health centre and enhanced primary care, for people released from custody with a chronic health condition or > 50 years [33]. The study suggested that the enhanced primary care program reduced criminal justice system costs, with an estimated a 12-month return of $2.55 to the state for every invested dollar [33].

### Where are the knowledge gaps in this area?

The review suggests knowledge gaps in this area relate to the location of studies. The included study locations were Canada, Australia, USA and The Netherlands. There were no studies from the United Kingdom, or Europe or low and middle income (LMIC) countries. More research is needed from other countries to make findings more generalisable. This review focused on primary care use and the search strategy incorporated terms around general practice, family practice, in addition to other community care for example, nurse practitioners, community pharmacy services, community dentistry and optometrists. Few studies were performed on the provision of care outside of general practice. Additional studies examining access to different healthcare services in the community among people after release from prison would give a clearer idea of challenges during transition from prison to community care. Table 5 describes the recommendations for further research in this area. Since most studies have been published in recent years, it was decided a posteriori to map the recommendations for future research (this was not a direct research question in this scoping review, and therefore was not part of the charting process of the included studies).

**Table 5:** Recommendations for further research

First author, year	Recommendations for further research
Calais‑Ferreira et al. 2022 [30]	Not reported
Carroll et al. 2017 [31]	Research on continuity of care and health outcomes after release from prison, further work to understand the sharing of information between health care settings in the prison and community
Dirkzwager et al. 2021 [32]	Around generalisability of findings i.e. more studies required in other countries. Research examining the length of custodial period, and the heath care use in prison in relation to the community setting. e.g. health before and after prison with information on physical and mental health care in prison
Harvey et al. 2022 [33]	Exploring the long-term costs of primary care programs for people during the transition from prison to community, including programs for health conditions and treatment options, e.g. substance use disorder or preventative care such as cancer screening
Howell et al. 2016 [34]	Interventions targeting people who have been incarcerated to improve blood pressure control and cardiovascular outcomes
Khanna et al. 2019 [35]	Interventions to facilitate linkage to care, other interventions such as discharge planning
Kouyoumdjian et al. 2018 [13]	Examining reasons for healthcare attendance (both in prison and post-release), care experiences such as preventive care and quality of care and collaborating with people with a lived experience of imprisonment in research. Interventions to improve access to care and quality of health care in prison and post-release
Kouyoumdjian et al. 2019 [12]	Investigation to define barriers to access to primary care and qualitative work with people who have been incarcerated and with primary care providers. Work supporting the linkage to high-quality primary care after prison release
Kouyoumdjian et al. 2020 [36]	Investigation of the experiences of hospitalisation among the prison population and for individuals after release, particularly in relation to lower rates of readmission among people who have been incarcerated. Investigation of barriers to hospital access, and methods of optimising primary care and emergency department care for the requirements of this population
Mahentharan et al. 2021 [37]	Research on the needs of people diagnosed with schizophrenia and specific risk factors for reincarceration
McConnon et al. 2019 [38]	Addressing health promotion and healthcare needs, for example via preventive care, coordination with population cancer screening programs and primary care services in the community, and training healthcare providers in preventive care of prison groups
Norris et al. 2021 [39]	Qualitative studies to better understand health care use disparities
Palis et al. 2022 [40]	Investigating methods to improve access to mental health services after release from prison
Palis et al. 2022 [41]	Investigating services for people with opioid use disorder, including opioid agonist treatment in prison and continuity in the provision of care after release
Wang et al. 2012 [42]	Early interventions addressing the transition of health care from prison to the community and engagement with community health workers before prison release
Wang et al. 2019 [43]	Investigating a wider range of outcomes, for example, across health and social service systems, and examining patient quality of life and well-being of individuals released from prison
Young et al. 2015 [44]	Using administrative health data to investigate a causal relationship between early primary care contact and increased utilisation of health services

## Discussion

This scoping review identified and collated the most recent evidence from data-linkage studies about primary care use after release from prison; and mapped the evidence from the included studies by methodology, key findings and gaps in research. Evidence in this research area is growing with most of the included studies published since 2019. Included studies were conducted across four countries, and most used cohort designs to investigate primary care visits (for example, use, time to first contact and quality of care). The reporting of the descriptive characteristics of each sample varied across the included studies. Reporting and analysing socio-demographics in this study population is important to help identify patient groups who are engaging (or not) with primary care. For example, approximately half of the included studies reported age, most (*n* = 14/17) reported race/ethnicity, but all reported gender/sex (Appendix 4). In the included studies, most data sources were linked was by unique identifiers. The study period after release from prison varied between the included studies, however the most common time periods examined were one- or two-years follow-up. Although most studies did consider methods to address re-incarceration, different approaches were used for example, excluding time spent in prison during re-incarceration from follow-up, censoring follow-up at re-incarceration, using re-incarceration as a study outcome, measuring the study exposure at each release from prison and using truncation for re-incarceration(s) during the follow-up period. Re-incarceration has potential methodological issues for the study of primary care use i.e. by varying the access an individual has to primary health care services in the community. Furthermore, health-related factors have been identified as risk factors for re-incarceration in analyses adjusting for demographic and criminal justice factors [45]. Therefore, the differences in methods used to address re-incarceration may pose important consequences on the reported outcomes. Few included studies reported using a checklist or technique which allows transparency in methodological and reporting quality. With an apparent lack of use of reporting guidelines in this area, more rigorous documentation would help improve future evidence synthesis and is recommended.

There is mixed evidence regarding levels of primary care use for people after prison release (compared to the general population), for example, with variations reported among studies such as higher and lower use [12, 13, 31]. The review found higher primary care use among specific patient groups after release from prison, for example, history of risky opiate use, diagnosis of a mental health condition or medication in prison [31], individuals living with HIV [35] and people with schizophrenia [37]. Females who have been incarcerated had higher morbidity and specific psychiatric conditions compared with males released from prison and females in the general population, and higher rates of primary care use after release from prison [39]. Therefore, suggesting that high primary care attendance relates to a greater demand for health care within patient groups. The suggested higher rates of contact may allow health professionals to identify other health risks or opportunities for health promotion in this marginalised group. A positive finding from this review indicated good connectivity in that contact with primary care services early after release from prison increased health service use, generally [44]. However, there is evidence of low numbers of people accessing primary care during the first month post-release (e.g. one study reported that in the first month after release, around two-thirds of women and three-quarters of men did not access primary care) [13]. Timely access to primary care also includes medication dispensed after release from prison, for example, a study suggested that approximately one-quarter of individuals with an opioid use disorder received opioid agonist treatment dispensation within two days of release from prison [41].

Multimorbidity (two or more chronic conditions) is one of the major challenges facing developed health care systems; with risk factors including increasing age, lower socioeconomic status, living in more deprived areas and health behaviour (such as alcohol use, smoking and low physical activity) [46, 47]. There is a high prevalence of multimorbidity among prison population [30]. There was increased primary care contact for people with moderate or complex multimorbidity in the two years after release from prison compared to having no multimorbidity, and the quality of care was found to be largely inadequate for moderate multimorbidity, but adequate in people with complex multimorbidity [30]. Understanding patterns in multimorbidity in people leaving prison and health disparities, may help address issues in quality of care. There were lower levels of colorectal and breast cancer screening among people released from custody compared to the general population [38]. This finding would suggest suboptimal care for people in prison in regard to screening and potentially missed opportunities for the early detection of cancer and the provision of services for this vulnerable population. Some benefits from enhanced primary care programmes after release from prison were reported, for example, a reduction in reincarceration [43] and criminal justice system costs [33] and research into improving the transition from health care prison to the community is an important step to improving outcomes. The studies included in this review compared enhanced primary care via the Transitions Clinic Network (TCN) programme to individuals not exposed to the programme [33, 43]. The TCN programme facilitates primary care clinics to increase health care services access, improve health status, and lower reincarceration rates among people recently released from prison with chronic health condition(s) or are over 50 years old [18, 33]. The enhanced primary care model in the TCN consists of specialist community health workers with a history of incarceration (i.e. having lived in prison and had their own post-release experiences) as part of a primary care team addressing the needs of people during the transition from prison to community living [33, 43]. The TCN programme provides care for health conditions, including substance use disorders and mental health conditions, and addresses social determinants of health such as housing, food access and employment after release from prison [33, 43]. Patient navigation has been investigated in the general population and among people release from prison. For example, a scoping review of all patients without a regular source of primary care (provider/practice), i.e. general population, included 20 studies (published between 2000 and 2016) and found that patient navigators, i.e. a person/process facilitating a linkage between an individual requiring primary care and a primary healthcare provider, may improve access to primary care and patient-centred care across different healthcare settings [48]. A separate scoping review of interventions connecting individuals to substance use services after release from jail (including 14 studies; published between 2001 and 2021), found that four of the included studies used peer or patient navigation as the core intervention component [49].

### Study strengths and limitations

This scoping review has several strengths. To our knowledge, this is the first review of record linkage studies about primary care utilisation after release from prison. The methods used follow the framework provided by Arksey and O’Malley and guidance by the Joanna Briggs Institute (JBI) [15, 16]. Other than additions to the data charting form, there have been no changes to the methods published in the study protocol [14]. This scoping review focused on data-linkage studies within the broader topic of primary care use after prison release, therefore findings specifically relate studies using linked data sources, and the results may be used to guide future research in this area. This scoping review has some limitations. The search strategy in this review used terms relating to ‘former’ incarceration to increase specificity, however, this may have reduced sensitivity and some publications may have been missed. Similarly, the search strategy used terms for general/family practice and other community healthcare services such as pharmacy, dentistry and optometry. It is possible that some studies may have been missed given the range of terms used across countries to describe primary care and related services. For example, a study by Sutherland et al. 2015 [50] was not included in our 1,050 publications screened by title and abstract in step 1 (i.e. publications identified by our search strategy) (Kinner, personal communication). The study (based in Australia) included 251 women (18–49 years) and used in prison baseline survey data (2008–2010) and prison medical records, linked to Pharmaceutical Benefits Scheme (PBS) claims data after release from prison (probabilistic linkage) [50]. The study reported dispensing of contraceptive medications within 30, 90 and 180 days of release from prison [50]. The study reported that contraceptive medication had been dispensed to 5 women (2%) at 30 days after release from prison; 9 women (4%) at 90 days after release, and 19 women (7.6%) by 180 days after release [50]. The specific health needs of women after release from prison highlights an important area for both research and policy for the provision of reproductive health in prison and after release. A search of publications by key authors in the field (as part of the search strategy) may have identified additional publications, however this is not a normal part of the scoping review process. The review did not include a search of the grey literature and was limited to publications available in English due to resources. Finally, the included studies were conducted in Canada, Australia, USA and The Netherlands, therefore impacting the generalisability of the review findings.

### Future research

The United Nation’s 2030 Agenda for Sustainable Development set goals and actions for people, planet and prosperity between 2015 and 2030 [51]. Although the health of the prison population is not reflected in the Sustainable Development Goal (SDGs) 2030, it has been advocated that improving the health of people in prison may directly/indirectly contribute to 15 of the total 17 SDGs [52]. The World Health Organisation defines that primary health care should be people centred, supporting needs such as health promotion, disease prevention, treatment, rehabilitation and palliative care [53]. More research is needed to examine whether all aspects of primary health care are being met after release from prison. Since most studies included in this review have been published in recent years, the recommendations for future research suggested by each paper have been summarised. This offers an opportunity to address the gaps in knowledge. In summary, the included studies suggested further research on health during all stages of an individual’s prison journey (including determining reasons for healthcare attendance and studies/interventions for specific conditions). More research is needed on the continuity of care, across all health and social service systems, and health outcomes after release from prison, and there is a need to address health promotion in the prison population, for example population cancer screening programs. Further work to understand the sharing of information between health care settings in the prison and community and methods around discharge planning could help identify challenges/solutions regarding the timeliness of primary care access. More work is required around primary care programmes after release from prison, e.g. engagement with community health workers and the long-term costs and benefits of primary care programs. The involvement of people with a lived experience of incarceration in research, including qualitative work, would provide better insight into the quality of life and well-being of individuals after release from prison. Finally, more studies are required across different countries to allow generalisability of findings.

Epidemiological population-based studies linking incarceration records and health care data can improve understanding around patterns of health care use, patient pathways such as people most at risk of not engaging with community care, facilitators/difficulties in accessing services, and health-related and other outcomes, to help profile people after release from prison. There is a need for research about aspects of pre-release healthcare management, for example, how to improve communication between prison and community healthcare providers and services in a transparent and accountable way that would facilitate early contact and the delivery of support after release; and to tap into the potential to implement innovative processes that would improve the transition of care for patient groups. The variant of the National Health Service (NHS) in each United Kingdom devolved nation is responsible for the organisation, provision and delivery of healthcare services (and in the case of Northern Ireland, for health and social services in Northern Ireland) for people in prison and in the community; and this arrangement presents an opportunity to understand and improve the connectedness of patient pathways from prison to community care services. Evidence synthesis of interventions to improve the health of people during incarceration and the first year after release has been reported. For example, a systematic review of randomised controlled trials included 95 studies [focused on substance abuse (*N* = 35), mental health (*n* = 28), infectious diseases (*n* = 18), health service use (*n* = 12) and chronic diseases (*n* = 2)] [54]. In most of the studies, the intervention was implemented during incarceration and the outcome was measured in the community after release [54]. Of the included interventions, 59 reported improved outcomes, and in 42 intervention studies outcomes were measured in the community after release [54]. Furthermore, linking data to interventions can be a strategy for loss to follow-up of marginalised populations, for example, by testing for selective biased attrition in trials and using record-linkage to administrative data for determining biased attrition [25].

## Conclusions

This scoping review identified evidence from data-linkage studies about primary care use after release from prison. Most studies included in this scoping review were published in recent years and are largely cohort study designs. The review suggested mixed evidence regarding levels of primary care use for people after prison release and has highlighted major challenges and areas of suboptimal care. Recommendations for further research have been discussed.

### Electronic supplementary material

Below is the link to the electronic supplementary material.

Supplementary Material 1

Supplementary Material 2

## Data Availability

Availability of data and materialsNot applicable. A data charting form used for data extracted from the full texts publications has been provided as supplementary material (Appendix 3).
