# Supplementary material for: Data linkage studies of primary care utilisation after release from prison: a scoping review
Source: BMC Prim Care. 2024 Aug 7;25:287. doi: 10.1186/s12875-024-02527-w (PMC11308621; doi:10.1186/s12875-024-02527-w)
Supplement: Supplementary file 2 — Supplementary Material 2 [file 12875_2024_2527_MOESM2_ESM.docx]

**Title**

**Data linkage studies of primary care utilisation after release from prison: a scoping review**

**Authors**

Janine A. Cooper ^1,2^*, Siobhán Murphy ^1,2^, Richard Kirk ^3^, Dermot O’Reilly ^1,2^, Michael Donnelly ^1,2^

^1^ Centre for Public Health, Queen's University Belfast, Royal Hospitals Site, Grosvenor Road, Belfast, UK

^2^ Administrative Data Research Centre Northern Ireland (ADRC NI), Centre for Public Health, Queen's University Belfast, Royal Hospitals Site, Grosvenor Road, Belfast, UK

^3^ South Eastern Health and Social Care Trust, Ulster Hospital, Dundonald, UK

*Corresponding author

Corresponding author contact: Cooper JA, Centre for Public Health, Queen's University Belfast, Royal Hospitals Site, Grosvenor Road, Belfast, UK. Administrative Data Research Centre Northern Ireland (ADRC NI), Centre for Public Health, Queen's University Belfast, Royal Hospitals Site, Grosvenor Road, Belfast, UK. Email: [j.cooper@qub.ac.uk](mailto:j.cooper@qub.ac.uk)

**Appendix 1 The Preferred Reporting Items for Systematic reviews and Meta-Analyses extension for Scoping Reviews checklist**

**Data linkage studies of primary care utilisation after release from prison: a scoping review**

Uploaded separately as supplementary material.

**Appendix 2 Search strategies for MEDLINE, EMBASE and Web of Science Core Collection**

Ovid MEDLINE(R) ALL

1 General Practice/ 15325

2 Family Practice/ 66933

3 General Practitioners/ 10419

4 GP.mp. 49054

5 Physicians, Family/ 17161

6 Primary Health Care/ 90730

7 primary care.mp. 143327

8 home visit*.mp. 10767

9 House Calls/ 4143

10 Family Health/ 24149

11 Physicians, Primary Care/ 4315

12 Nurse Practitioners/ 18785

13 Community Pharmacy Services/ 5693

14 Community Dentistry/ 1233

15 Optometrists/ 216

16 Optometry/ 5722

17 Prisoners/ 18273

18 Prisons/ 11087

19 after release.mp. 4490

20 prison release.mp. 157

21 release* from prison.mp. 609

22 ex-prisoner*.mp. 214

23 recently released.mp. 2144

24 1 or 2 or 3 or 4 or 5 or 6 or 7 or 8 or 9 or 10 or 11 or 12 or 13 or 14 or 15 or 16 350106

25 17 or 18 or 19 or 20 or 21 or 22 or 23 31107

26 24 and 25 453

27 limit 26 to (english language and humans and yr="2012 -Current") 189

Embase

1 general practice/ 84370

2 family practice.mp. 10362

3 general practitioner/ 116783

4 GP.mp. 96372

5 family medicine/ 13228

6 family physician*.mp. 21025

7 primary health care/ 78080

8 primary care.mp. 198088

9 primary medical care/ 130114

10 home visit/ 4948

11 home care/ 69242

12 house call*.mp. 942

13 family health/ 11565

14 primary care physician*.mp. 31828

15 nurse practitioner/ 27986

16 "pharmacy (shop)"/ 11665

17 community pharmacist/ 2640

18 community pharmac*.mp. 18128

19 community dentist.mp. 8

20 dentist/ 27353

21 optometry/ 6654

22 optometrist/ 1303

23 prisoner/ 18589

24 prison/ 15194

25 after release.mp. 5161

26 prison release.mp. 179

27 release* from prison.mp. 708

28 ex-prisoner*.mp. 231

29 recently released.mp. 2896

30 1 or 2 or 3 or 4 or 5 or 6 or 7 or 8 or 9 or 10 or 11 or 12 or 13 or 14 or 15 or 16 or 17 or 18 or 19 or 20 or 21 or 22 646399

31 23 or 24 or 25 or 26 or 27 or 28 or 29 36616

32 30 and 31 979

33 limit 32 to (human and english language and yr="2012 -Current") 523

**Database**: Web of Science Core Collection

**"general practice" or "family practice" or "general practitioner*" or gp or "family medicine" or "family physician*" or "primary health care" or "primary care" or "primary medical care" or "home visit*" or "house call*" or "home care" or "family health" or "primary care physician*" or "nurse practitioner*" or "pharmac* shop*" or "community pharmac*" or "community dentist*" or optometrist* or optometry** (All Fields) and **prisoner* or prison* or "after release*" or "prison release" or "release* from prison" or ex-prisoner* or "recently released"** (All Fields) and **2012** or **2013** or **2014** or **2015** or **2016** or **2017** or **2018** or **2019** or **2020** or **2021** or **2022** or **2023** (Publication Years) and **English** (Languages)

**Appendix 3 Data charting form used in review**

**Data linkage studies of primary care utilisation after release from prison: a scoping review**

**Data charting form**

**General Information**

|  | **Information as stated in publication** |
| --- | --- |
| **Initials of researcher completing the data charting form** |  |
| **Date of completion of data charting form** |  |
| **First author (i.e. lead author on paper)** |  |
| **Year of publication** |  |
| **Study title** |  |
| **Journal name** |  |

**Methods**

|  | **Information as stated in publication** |
| --- | --- |
| **Study design** |  |
| **Sources of linked data** |  |
| **Custodial setting (e.g. prison, jail)** |  |
| **Primary care service(s) (e.g. general practice, pharmacy, dentist, optician)** |  |
| **Method of data linkage** |  |
| **Years of data linked** |  |
| **Time period examined after prison release** |  |
| **Re-incarceration after release** |  |
| **Source of comparator data** |  |
| **Matching** |  |

**Participants**

|  | **Information as stated in publication** |
| --- | --- |
| **Setting location (country)** |  |
| **Number of participants in cohort/number of cases** |  |
| **Number of controls/number in comparator group** |  |
| **Inclusion of participants** |  |
| **Exclusion of participants** |  |
| **Age (state time point e.g. release)** |  |
| **Gender** |  |
| **Race/ethnicity** |  |

**Outcomes**

|  | **Information as stated in publication** |
| --- | --- |
| **Primary and secondary outcomes (as reported)** |  |

**Statistical analysis**

|  | **Information as stated in publication** |
| --- | --- |
| **Statistical analysis (as reported)** |  |

**Results**

|  | **Information as stated in publication** |
| --- | --- |
| **Results (for outcomes reported)** |  |

**Quality assessment**

|  | **Information as stated in publication** |
| --- | --- |
| **Quality assessment checklist or technique (state if use was reported)** |  |

**Appendix 4 Additional characteristics of included studies**

| **Author, year** | **Sources of linked data** | **Source of comparator data** | **Matching** | **No. of participants/cases** | **No. of controls/ number in comparator group** | **Age** | **Gender** | **Race/ ethnicity** |
| --- | --- | --- | --- | --- | --- | --- | --- | --- |
| Calais‑Ferreira  2022 [30] | In-prison survey data and coded prison medical records linked with Medicare, National Death Index (NDI), correctional services. | n/a | n/a | 1046 | n/a | 18+ years (self-report) | Sex (male/ female) (self-report) | Indigenous status (self-report) |
| Carroll  2017 [31] | Baseline interviews, correctional institution records, prison medical records. Exposure to hepatitis C virus (HCV) (self-report of exposure or positive HCV antibody test in prison medical records). Medicare Benefits Schedule (MBS) claims data. Pharmaceutical Benefits Scheme (PBS) claims Queensland Corrective Services (QCS). National Death Index (NDI). | General Queensland population | Same sex and age group | 1190 | Not stated | 18+ years | Sex  (men/ women) | Indigenous Australians |
| Dirkzwager  2021 [32] | System of Social Statistical Datasets (SSD) (Statistics Netherlands). NIVEL Primary Care Database (NIVEL-PCD) includes data from the electronic health records (EHRs) of a representative national sample (about 7%) of all general practices in the Netherlands. Dutch National Prison Database from Statistics Netherlands. | General population, patients registered in NIVEL-PCD and not detained in 2014/2015. | 5:1. Matched on same general practice, age (+/- 1 year), sex. | 952 | 4760 | Age 16+ years (age groups 17-25; 26-35; 36-46; 47+ years) | Sex  (male/ female) | Not stated |
| Harvey  2022 [33] | Connecticut Department of Correction. Connecticut Medicaid. Connecticut Department of Mental Health and Addiction Services. | Individuals released from prison to another medium-sized city in Connecticut (without TCN care at time of study) | Estimated propensity scores (32 covariates) and greedy matching algorithm without replacement to create a 1:1 case-control sample | 94 | 94 | Not clear | Gender. Not clear | Race/ ethnicity (White, Black, Hispanic) |
| Howell  2016 [34] | Self-reported, administrative, and clinical data from 8 Veterans Health Administration (VHA) clinical sites. | Not stated | n/a | Incarceration: 163 (7%) reported recent and 904 (39%) reported past history | n/a | Not stated | Sex (male/ female) | Race/ ethnicity (Black, Non-black) |
| Khanna  2019 [35] | Data from the Ontario Ministry of Community Safety and Correctional Services (MCSCS). Ontario’s administrative health records (ICES). Linked persons in the MCSCS dataset to health databases. Registered Persons Database (Ontario’s registry of eligibility for health insurance). Ontario HIV database. OHIP database for primary care and non-primary ambulatory care visits. National Ambulatory Care Reporting System (NACRS) for emergency department visits. CIHI-Discharge Abstracts Database (DAD). Ontario Mental Health Reporting Systems (OMHRS) for hospitalizations. | Three comparator groups: people with no HIV released from provincial prison, people with HIV in the general population, and people with no HIV in the general population | 3:1 matching age group, sex, eligibility for OHIP coverage | 330 | 990 HIV-negative persons released from provincial prison in 2010, 990 people with HIV and no history of incarceration and 990 HIV-negative persons in the general population. | Not stated | Sex | Race (self-report) |
| Kouyoumdjian  2018 [13] | Ministry of Community Safety and Correctional Services (MCSCS). Registered Persons Database. ICES, Ontario Ministry of Health and Long-Term Care, Health administrative data. | General population (Registered Persons Database) | 4:1. Matched on age, sex | 48,861 | 195,444 | ≥18 years | Sex (male/ female) | Race (self-report) (Missing, White, Black, Aboriginal, Other) |
| Kouyoumdjian  2019 [12] | Ontario Ministry of Community Safety and Correctional Services (MCSCS) (data on provincial prison). Health administrative data for Ontario residents. Registered Persons Database (RPDB), OHIP database, National Ambulatory Care Reporting System, Discharge Abstract Database, Community Health Centre (CHC) database and Ontario Mental Health Reporting System. | General population group | 4:1. Matched on age, sex. Registered Persons Database (RPDB), registered for Ontario Health Insurance Plan (OHIP) coverage | 48,861 | 195,444 | Adults | Sex (male/ female) | Race (self-report) |
| Kouyoumdjian  2020 [36] | Ontario Ministry of Community Safety and Correctional Services, health administrative data at ICES, Canadian Institute for Health Information Discharge Abstracts Database, Ontario Health Insurance Plan-eligible people in the Registered Persons Database, National Ambulatory Care Reporting System, data from the Ontario Health Insurance Plan data on primary care use | General population - Ontario Health Insurance Plan-eligible people in the Registered Persons Database | 1:1. Matched on age, sex, hospitalisation case mix group, hospital discharge year | 262 (hospitalisations for people in prison) | 1,268 (hospitalisations for people recently released from prison) | Adults | Sex | Race (self-report) Aboriginal, Black, White, Other. Race not available for general population |
| Mahentharan  2021 [37] | Ministry of Community Safety and Correctional Services dataset. Canadian Institute for Health Information Discharge Abstract Database (CIHI DAD), Ontario Mental Health Reporting System (OMHRS), National Ambulatory Care Reporting System (NACRS), OHIP Claims Database. The Registered Persons Database (RPDB) (for population and demographic data) (Ministry of Health and Long-term Care in Ontario). | None stated | n/a | 46,928 | n/a | ≥18 years | Sex (male/ female) | Race (self-report) Missing, Aboriginal, Black, East Asian, Hispanic, South Asian, South East Asian, West Asian/Arabic, White, Other racial origin, Declined to specify, Racial origin unknown |
| McConnon  2019 [38] | Ontario Ministry of Community Safety and Correctional Services, Ontario Health Insurance Plan (OHIP) (public health insurance plan, pays for healthcare services including primary care and colorectal and breast cancer screening tests for Ontario residents). Ontario Breast Screening Program database. ICES (Ontario Ministry of Health and Long-Term Care) health administrative data for Ontario residents. | General population in Ontario, Canada. | n/a  OHIP-eligible on July 1, 2010 in the ICES registry of OHIP-eligible individuals, excluding people in the corrections group | Colorectal cancer screening N=3,803 and breast cancer screening N=249 | General population (colorectal cancer screening N=2,757,584 and breast cancer screening N=1,099,942) | >50 years (index date) | Sex (male/ female) | Race (self-report) Aboriginal, Black, White, Asian, Hispanic, Other. No individual-level race data available for general population |
| Norris  2021 [39] | Ontario Health Insurance Plan (OHIP) system. Correctional data. Registered Persons Database. Ambulatory care data (OHIP). Emergency department data (National Ambulatory Care Reporting System, or NACRS), hospital admissions data (Canada Institute for Health Information Discharge Abstract Database, or DAD, and Ontario Mental Health Reporting System, or OMHRS) | Registered Persons Database | Four age-matched females in the general population. | Female incarceration group (N = 6,107) | Male incarceration group, N=42,754. Female general population group, (N=24,428) | Adults | Sex | Race (self-report) Unknown Aboriginal Black White Other. Race data not available for female general population |
| Palis  2022 [40] | British Columbia Provincial Overdose Cohort (BC-ODC). BC-ODC contains linked administrative health and corrections data. Mortality data from Vital Statistics Agency. Solicitor General’s records of release from BC’s 10 provincial correctional centers, Ministry of Social Development and Poverty Reduction records | None stated | n/a | 1664 | n/a | Adults (≥18 years) | Biological sex (male/ female) | n/a |
| Palis  2022 [41] | British Columbia Provincial Overdose Cohort (BC-ODC). BC-ODC - linked health and corrections records. OAT dispensation from PharmaNet records (provincial drug dispensation database) | None stated | n/a | 13,380 | n/a | Not stated | Biological sex (male/ female) | Not stated |
| Wang  2012 [42] | Electronic repository (safety-net health care and jail health systems). Baseline questionnaire. UCSF Clinical and Translational Science Institute’s The Health Records Electronic Data Set (THREDS) and the Jail Health Services database: electronic health record and registration system. Mortality data from the California Department of Health Services Death Registry. Jail Health Services database. | Randomised to expedited primary care in safety net clinic | n/a | 98 | 102 | None stated | Gender | Race and ethnicity (Asian, Black, Hispanic, Native American, White) |
| Wang  2019 [43] | Administrative data of Connecticut Department of Correction, Department of Mental Health and Addiction Services, Department of Social Service, Court Support Services Division, and Department of Public Health | People released from prison during the same period (to urban area similar to location of TCN programme) | Greedy matching algorithm. 1:1. Propensity score matching. | 94 | 94 | Not stated | Gender | Race/ ethnicity  (White, Black, Hispanic) |
| Young  2015 [44] | Baseline interview (within 6 weeks of expected release). Three follow-up telephone interviews conducted approx. 1, 3 and 6 months after release. Prison medical records. Queensland Correctional Service (QCS) records. Australian National Death Index. | None stated | n/a | 847 | n/a | ≥18 years | Gender (male/ female) | Indigenous status |

**Appendix 5 Study outcomes and statistical analysis in included studies**

| **Author, year** | **Primary and secondary outcomes (as reported)** | **Statistical analysis (as reported)** |
| --- | --- | --- |
| Calais‑Ferreira et al. 2022 [30] | Measures of quality of primary healthcare included (a) continuity of care, and (b) use of extended consultations. Outcomes were Usual Provider Continuity Index (UPCI), Continuity of Care (COC) Index, and having at least one extended primary care consultation (>20minutes). | Descriptive statistics. Unadjusted rates of primary care contact post-release. Logistic regression to examine the association between multimorbidity (no multimorbidity, moderate, complex), rate of primary care contact, and quality of care (COC and UPCI, at least one extended consultation), adjusting for social determinants of health (age, sex, Indigenous status, unstable accommodation, unemployment. Sensitivity analyses. |
| Carroll et al. 2017 [31] | Rates of general practice attendance during the two years after prison release. | Crude rates of GP attendance per person-year. Compared with general population rates, and age- and sex-standardised rate ratios (SRRs) calculated, using indirect standardisation. Negative binomial regression to identify characteristics associated with higher rates of GP contact. Unadjusted incidence rate ratios (IRRs) were calculated (95% confidence intervals). Multivariate model (backwards elimination of covariates P > 0.05). |
| Dirkzwager et al. 2021 [32] | Health problems one-year pre- and post-prison. Health problems (dichotomous) i.e. referring to ‘attending a GP for a specific health problem ≥1 times that year’ and ‘not presenting that health problem or did not visit their GP at all during that year’. | Prevalence rates calculated based on health problems. Differences in socio-demographic characteristics. Odds ratios (ORs) with 95% confidence intervals (Cis), testing (a) differences in pre-detention prevalence rates (detainees/controls), and (b) the change in health problems from pre- to post-detention. Ratios (and 95% CIs) of the ORs comparing the pre- and post-detention rates of detainees and the ORs comparing the pre-and post-detention rates of controls calculated. Logistic regression analyses (pre-detention health differences between detainees/controls). |
| Harvey et al. 2022 [33] | Not clear: Costs: (1) associated with the Transitions Clinic Network (TCN) program; (2) by Medicaid/criminal justice system. | Included t-tests and chi-square tests. Associations (Transitions Clinic Network (TCN) program participation and Medicaid/criminal justice system costs) over 1 year using bivariate analyses with nonparametric bootstrapping method. Sensitivity analysis. |
| Howell et al. 2016 [34] | Measured BP control using clinical data collected in the 12 months following the survey. Measured primary care engagement (defined as ≥2 primary care visits at least 90 days apart in the 12-24 months prior to the survey and, in the 12 months after the survey). Measured antihypertensive medications receipt in the 12 months after the survey (classification codes in the pharmacy refill data), and calculated the medication possession ratio (percentage of days with antihypertensive medication). | Descriptive statistics. Logistic regression, examining the association between incarceration history and uncontrolled BP. Mediation analyses. |
| Khanna et al. 2019 [35] | Rates of primary care use. | Calculated the rates and 95% confidence intervals for primary care. Calculated rate of use based on person-time at-risk and the number of visits for primary care. Kaplan-Meier method to calculate time to access any ambulatory care for people with and without HIV (defined ambulatory care as primary or specialty care but not emergency department care). |
| Kouyoumdjian et al. 2018 [13] | Primary care use for persons released from prison (compared to use in the general population). | Compared primary care use rates at various time periods after release from prison. Calculated rate ratios for primary care use (prison release group compared to general population). Used generalized estimating equations. Examined the proportion of people using primary care (chi squared tests) and time to first primary care visit post-release (Kaplan-Meier). |
| Kouyoumdjian et al. 2019 [12] | Primary care attachment and team-based primary care attachment:  Baseline period was 2 years before prison.  Follow-up period was 2 years after release. | Proportions attached to primary care and team-based primary care (compared using χ2 tests). Primary care attachment and team-based primary care attachment (as percentage) included prison release, and specific chronic conditions/aggregated diagnosis groups. |
| Kouyoumdjian et al. 2020 [36] | 30-day medical-surgical readmission to hospital. Access to care (including primary care) after hospital discharge. | Compared groups (people in prison, people recently released from prison and general population). Kaplan-Meier method to calculate the frequency of readmission. Stratified log-rank tests to compare the risk in groups. Cox survival analysis to assess the unadjusted association between imprisonment status and readmission. |
| Mahentharan et al. 2021 [37] | Primary outcome was time to reincarceration.  Secondary outcomes included measures of correctional events and health service use. For primary care: time to first primary care physician (PCP) contact (including total, mental health–related, and non-mental health–related contact) after release. | Health service use encounters: calculated 3-year period before index incarceration and 5-year after the index release. Health care use was measured during the index incarceration. Descriptive data were summarised. Survival analysis for the association between schizophrenia and reincarceration. Cox proportional hazards model. |
| McConnon et al. 2019 [38] | Screen-eligible individuals overdue for breast and colorectal cancer screening. | Calculated frequencies. Chi-square tests and log binomial regression models to compare the proportion of individuals overdue for screening on admission or on July 1, 2010 for the general population, and still overdue after 3 years. Calculated the proportion of any primary care encounters, and the median and IQR for the number of primary care encounters in the 3 years before and 3 years after the index date. |
| Norris et al. 2021 [39] | Primary care visits. | Calculated health care use rates for each health care type and period. Calculated mortality rates. Standardized differences. Calculated unadjusted rate ratios for mortality rates and for each type of health care use. |
| Palis et al. 2022 [40] | Release to reincarceration (with/without mental health services access). Influence of timeliness of access to mental health services on subsequent reincarceration. | A multistate modeling approach. Cox proportional hazard models stratified by transitions. State arrival time was added to the stratified Cox proportional hazards models. Sensitivity analysis. |
| Palis et al. 2022 [41] | Community Opioid agonist treatment (OAT) dispensation within two days of release from prison. | Study characteristics. Generalized estimating equation (GEE) was used to estimate the odds of OAT dispensation within two days post-release (adjusted for multiple releases for the same person). Unadjusted and adjusted odds ratios. Multivariable models. Post-hoc analyses, an interaction term between stimulant use disorder (StUD) diagnosis and mental illness was tested. Stratified odds ratios. Bonferroni correction was used to adjust for multiple comparisons. Sensitivity analyses. |
| Wang et al. 2012 [42] | Having two or more visits to the study-assigned primary care clinic. | Intention-to-treat analysis. Chi-2 test to compare primary care and incarceration between the two intervention groups. |
| Wang et al. 2019 [43] | Primary outcome of interest was reincarceration within 12 months from the index date. Secondary outcomes of this study included preventable emergency department visits, hospitalisations and length of hospital stays. | T-tests and χ2 analysis. Logistic regression and zero-inflated negative binomial regression models. |
| Young et al. 2015 [44] | Adjusted time-to-event hazard rates for hospital, mental health, alcohol and other drug and subsequent primary care physician service use (assessed as multiple failure time-interval data). Outcomes were self-reported use of mental health, alcohol and other drug, hospital, and subsequent primary care physician services in the community. Subsequent primary care physician (PCP) service use was examined at 3 and 6 months follow-up. | Descriptive statistics. Multivariate Andersen-Gill extension of a Cox proportional hazards model. |

**Appendix 6 Summary of results of included studies**

| **First author, year** | **Results (for outcomes reported)** |
| --- | --- |
| Calais‑Ferreira et al. 2022 [30] | Outcome: Usual Provider Continuity Index (UPCI), Continuity of Care (COC) Extended Primary Care Consultation.  The rate of primary care visits per person-year, after excluding time in prison and censoring for deaths, was 7.33 (95%CI 7.20–7.45) visits per person-year. Of 1046 participants 914, 87.4% had at least one primary care visit during follow-up. 620 (59.3%) had at least one extended consultation. 844 (80.7%) had two or more primary care encounters during follow-up. Women were more likely than men (191/233, 82.0% vs. 414/611, 67.8%) to have received an extended consultation (p<0.001).  Multimorbidity was present for 761 (73%) participants, being more prevalent among females (85%) than males (69%), and more common for participants aged ≥45years (134/159, 84.3%) than for younger groups.  Participants with moderate and complex multimorbidity had 2.6 (95%CI 1.9–3.6) and 4.5 (95%CI 3.2–6.4) times the odds of having a high rate of primary care contact (defined as nine or more contacts per person-year), compared to people without multimorbidity, respectively.  In fully adjusted models, moderate multimorbidity was not associated with UPCI or COC, but was associated with having at least one long consultation (>20 minutes) (AOR 1.64, 95%CI 1.14–2.39). Complex multimorbidity was positively associated with UPCI (AOR 1.83, 95%CI 1.20–2.80), COC (AOR 1.87, 95%CI 1.22–2.84), and having at least one long consultation (AOR 2.52, 95%CI 1.59–4.00). Indigenous status was negatively associated with UPCI (AOR 0.54, 95%CI 0.37–0.80) and COC (AOR 0.53, 95%CI: 0.36–0.77). Being <25 years was negatively associated with having a long consultation (compared to 25–44 years) (AOR 0.64 95%CI: 0.44–0.93). |
| Carroll et al. 2017 [31] | Outcome: GP attendance.  During the 2 years after release: Former prisoners attended GP services twice as frequently as the general population (SRR 2.04, 95%CI 2.00-2.07). The rate for men aged 25-34 years was 2.8 times that of other Queensland men of the same age (SRR 2.83, 95%CI 2.74-2.92). GP attendance by male former prisoners increased with age (consistent with trends in the general community). There was no consistent increase with age among women (participants or the general population); among female former prisoners, the rate was highest for those aged 25-34 years. 87% of participants had at least one contact with a GP. More women had at least one contact (94% women and 85% men). 42% of GP encounters resulted in a filled prescription. Encounters associated with diagnostic testing were nearly twice as common for young women as for older women or men of any age (21% v 11-12%).  In fully adjusted models: GP attendance rates were higher in participants; with a history of risky opiate use (IRR 2.09, 95%CI 1.65-2.65; P<0.001), having ever been diagnosed with a mental disorder (IRR 1.32, 95%CI 1.14-1.53; P=0.001), and receiving medication while in prison (IRR 1.82, 95%CI 1.58-2.10; P<0.001). GP attendance rate was lower in participants with a history of risky methamphetamine use (IRR 0.71, 95%CI 0.58-0.88; P<0.001). GP attendance rates were higher for older (per year) IRR 1.01, 95%CI 1.00-1.02; P<0.001) and females (IRR 1.35, 95%CI 1.15-1.56; P<0.001), but lower for Indigenous participants (IRR 0.76, 95%CI 0.64-0.89; P=0.001). |
| Dirkzwager et al. 2021 [32] | Outcome: Changes in health problems from pre- to post-detention  Males: Only for circulatory problems, a statistically significant change was observed. In the year after their detention, male detainees were somewhat more likely to report circulatory problems when compared with the year prior to their detention (16.8 vs. 12.9%, respectively; OR 1.36, 95%CI 1.04-1.79). Subsequently, the change in prevalence rates from pre- to post-detention was tested for detainees and controls. No statistically significant differences in changes in health problems between male detainees and controls were observed.  Females: The findings for the females mirror those for the males. No statistically significant changes in female detainees’ health problems over time. In addition, only for one group of health problems a significant difference was observed in the change in health problems between female detainees and controls. Female detainees differed from their matched controls with respect to changes in general and unspecified health problems (OR 1.92, 95%CI 1.05-3.53). While the prevalence of general and unspecified health problems increased over time for female detainees (from 38% to 49%), the prevalence of such health problems decreased for the female controls (from 31% to 27%). |
| Harvey et al. 2022 [33] | Outcome: Average monthly Medicaid costs per individual in transitions clinic network and matched comparison groups (shown for primary care, pharmaceutical and dental).  Primary care (includes services and visits provided by primary care providers) - Transitions Clinic Network (n=94) Mean: $324 and SD: $813 - Comparison Group (n=94) Mean: $381 and SD: $1422. Mean in Differences (95% confidence interval) −58(−394,245).  Pharmaceutical - Transitions Clinic Network (n=94) Mean: $528 and SD: $2243 - Comparison Group (n=94) Mean: $315 and SD: $952. Mean in Differences (95% confidence interval) 213 (−215,726).  Dental - Transitions Clinic Network (n=94) Mean: $34 and SD: $52 - Comparison Group (n=94) Mean: $31 and SD $54. Mean in Differences (95% confidence interval) 3 (−13,18). The 12-month TCN program estimated cost was at $54,394 ($146 per participant per month).  Average monthly Medicaid costs per participant were not statistically different between the TCN ($1737±$3449) and comparison ($1356±$2530) groups. Average monthly criminal justice system costs per participant were significantly lower among TCN group ($733±$1130) compared with the matched group ($1276±$1738, p<0.05). |
| Howell et al. 2016 [34] | Outcome: Primary care engagement  Primary care engagement after the survey did not meet the requirement of being associated with both the exposure (incarceration history) and outcome (uncontrolled hypertension).  *Baseline Characteristics of the Study Sample by Incarceration History (N = 2304).*  Recent incarceration, N=144, 88%, (total= 163).  Past history of incarceration, N=802, 89%, (total= 904).  Never incarcerated, N=1061, 86%, (total= 1237).  Participants with recent or past history of incarceration had higher levels of uncontrolled blood pressure (BP) than those without a history of incarceration (67 % vs. 56 % vs. 51 %, p < 0.001). At a bivariate level, recent (OR 2.01, 95%CI 1.42–2.85) and past history of incarceration (OR 1.26, 95%CI 1.06–1.49) were associated with uncontrolled BP in the year after the survey compared with participants without a history of incarceration. Primary care engagement post survey was also associated with higher levels of uncontrolled BP (OR 2.87 (2.22–3.72)).  After adjustment for age, HIV status, sex, race/ethnicity, educational attainment, income, body mass index and housing status, the association between recent incarceration and uncontrolled BP remained (adjusted odds ratio [AOR] 1.64, 95%CI 1.14–2.36). However, this did not remain for history of incarceration and uncontrolled BP (AOR 1.12, 95%CI 0.94–1.34).  Further adjustment for history of smoking, unhealthy alcohol use, illicit drug use, and primary care engagement prior to the survey found the association between recent incarceration and uncontrolled BP remained (AOR 1.57, 95%CI 1.09–2.26). |
| Khanna et al. 2019 [35] | Outcome: Primary care health care  *30 days post-release (visits per person year 95%CI)*  Prison release groups HIV positive (n=330) 15.9 (14.3–17.6); Prison release groups HIV-negative (n=990) 7.6 (6.9–8.2); General population HIV-positive (n=990) 6.1 (5.5–6.6); General population HIV-negative (n=990) 2.7 (2.4–3.1).  Rates of primary care utilization were similar for all time intervals, though significantly lower for people with HIV released from prison in the 365 days after release compared to the first 30 days.  *90 days post-release (visits per person year 95%CI)*  Prison release groups HIV positive (n=330) 13.9 (13.0–14.9); Prison release groups HIV-negative (n=990) 7.1 (6.9–7.6); General population HIV-positive (n=990) 5.8 (5.5–6.1); General population HIV-negative (n=990) 2.6 (2.4–2.8).  *365 days post-release* (visits per person year 95%CI): Prison release groups HIV positive (n=330) 13.0 (12.5–13.5); Prison release groups HIV-negative (n=990) 6.9 (6.7–7.1); General population HIV-positive (n=990) 5.9 (5.7–6.0); General population HIV-negative (n=990) 2.6 (2.5–2.7).  Kaplan-Meier analysis (after release from provincial prison): 24 days (95% CI 17–30) for 50% of people with HIV and 138 days (95% CI 104–179) for 75% of people with HIV to access any ambulatory care. 177 days (95% CI 136–239) for 50% of people with HIV to access HIV-specific ambulatory care. 50% of people with HIV in the general population accessed HIV-specific care by 154 days (95% CI 133–183) (data not shown). |
| Kouyoumdjian et al. 2018 [13] | Outcome: Primary care use rate (visits/person year) for persons released from prison by period relative to time in prison (same period control).  *In prison rates*: 12.2  *Post-release rates (days)*:  0-6: 9.1  7-29: 6.6  30-89: 6.0  90-179: 6.0  180-364: 6.3  365-730: 6.3  *confidence intervals not numerically reported  *Unadjusted and adjusted rate ratio of primary care utilization for persons released from provincial prison in 2010 and age and sex-matched general population controls in Ontario, Canada, by period relative to time in prison: Unadjusted rate ratio (95% CI):*  *In prison:* RR 6.1 (95% CI 5.9, 6.2).  *Post-release days*:  0-6: RR 3.7 (95% CI 3.6, 3.8)  7-29 : RR 2.6 (95% CI 2.6, 2.7)  30-89 : RR 2.4 (95% CI 2.4, 2.5)  90-179: RR 2.4 (95% CI 2.3, 2.5)  180-364: RR 2.4 (95% CI 2.4, 2.5)  365-730: RR 2.5 (95% CI 2.4, 2.6)  *Rate ratio adjusted for neighbourhood income quintile and rurality (95% CI):*  In prison: RR 6.1 (95% CI 5.9, 6.2).  Post-release days:  0-6: RR 3.8 (95% CI 3.7, 3.9)  7-29: RR 2.7 (95% CI 2.6, 2.8)  30-89: RR 2.5 (95% CI 2.4, 2.6)  90-179: RR 2.5 (95% CI 2.4, 2.6)  180-364: RR 2.5 (95% CI 2.4, 2.5)  365-730: RR 2.5 (95% CI 2.4, 2.6)  *Rate ratio adjusted for neighbourhood income quintile, rurality, and Aggregated Diagnosis Groups (95% CI):*  *In prison:* RR 3.9 (95%CI 3.8-4.0).  *Post-release days*:  0-6: RR 2.7 (95%CI 2.6-2.8)  7-29: RR 1.9 (95%CI 1.9-2.0)  30-89: RR 1.8 (95%CI 1.7-1.9)  90-179: RR 1.8 (95%CI 1.7-1.9)  180-364: RR 1.8 (95%CI 1.8-1.9)  365-730: RR 1.9 (95%CI 1.8-2.0)  First use of primary care after release from prison to two years: In the month after release, 66.3% of women and 75.5% of men had not accessed primary care. By three months after release, 50.5% of women and 62.9% of men had not accessed primary care. By two years after release, 16.8% of women and 28.2% of men had not accessed primary care.  The proportion of people who accessed any primary care was significantly higher for those in the prison release (PR) group compared to general population (GP) controls for the periods in prison and post-release days 0-6, 7-29 and 30-89. In contrast, the proportion that accessed any primary care was significantly greater for the general population compared to the prison release group for days 180-364 and 365-730 post-release.  *Proportion of persons released from provincial prison in 2010 and age- and sex-matched general population with any primary care utilization, by period relative to time in prison* (same period for controls):  *In prison:* PR n=48,861 40.8% v GP n=195,444 14.0% (<0.001)  *Post-release days*  0-6: PR n=48,861 13.4% v GP n=195,444 4.4% (<0.001)  7-29: PR n=47,870 19.5% v GP n=195,393 12.0% (<0.001)  30-89: PR n=44,939 28.5% v GP n=195,231 24.2% (<0.001)  90-179: PR n=39,328 33.3% v GP n=194,794 31.7% (<0.001)  180-364: PR: n=33,538 43.9% v GP n=194,158 47.3% (<0.001)  365-730: PR n=26,055 58.3% v GP n=193,040 61.8% (<0.001) |
| Kouyoumdjian et al. 2019 [12] | Outcome: Primary Care Attachment (PCA) and any team-based primary care attachment (APCA)  PCA  Baseline: PR 58.9% v GP 84.1% (P< .001):  Follow-up: PR 63.0% v GP 84.4% (P< .001).  APCA  Baseline: PR 14.4% v GP 16.1% (P< .001).  Follow-up: PR 19.9% v GP 21.6% (P<.001).  *Persons released from provincial prison in Ontario in 2010 (n=48,861) with PCA and APCA during the follow-up period, by chronic medical condition and comorbidity index*  Condition  *Diabetes* (n=2341) PCA 76.1%, APCA 24.8%  *Hypertension* (n=3629) PCA 76.3%, APCA 22.6%  *COPD (*n=2178) PCA 77.1%, APCA 27.8%  *Asthma* (n=8011) PCA 73.1%, APCA 22.8%  *HIV* (n=330) PCA 76.1%, APCA 33.0%  *Schizophrenia* (n=1909) PCA 73.2%, APCA 25.3%  *Anxiety disorders* (n=3757) PCA 74.8%, APCA attachment 28.3%  *Mood disorders* (n=3318) PCA 77.5%, APCA 27.7%  *Substance-related disorders* (n=8270) PCA 73.3%, APCA 26.9%  *No. of Aggregated Diagnosis Groups:*  0-4 (n=25383) PCA 51.5%, APCA 16.7%  5-9 (n=17395) PCA 73.9%, APCA 22.8%  ≥10 (n=6083) PCA 79.7%, APCA 24.5%. |
| Kouyoumdjian et al. 2020 [36] | Outcome: Primary care attendance post hospital discharge  Post-discharge care Primary care.  *7 days*  People in prison: 181 (69.1%) v General population 64 (24.4%): Standardized difference: 1.00.  Released from prison: 329 (25.6%) v General population 348 (27.1%): Standardized difference: 0.03.  30 days  People in prison: 224 (85.5%) v General population 125 (47.7%): Standardized difference: 0.87.  Released from prison: 637 (49.5%) v General population 677 (52.6%): Standardized difference: 0.06.  Hazard ratio (HR) for readmission by 30 days. Primary care: HR 0.74 (0.40–1.38) HR_adj_ 0.78 (0.60–1.02). |
| Mahentharan et al. 2021 [37] | (Supplementary material)  *Outcome: Number of Primary Care Physician visits (Median (IQR)):*  Individuals without Schizophrenia: 10 (3-28), Individuals with Schizophrenia: 19 (7-43), All Persons: 11 (3-29); Standardized Difference: 0.38  *Number of MH visits to PCP (Median (IQR):*  Individuals without Schizophrenia: 1 (0-6), Individuals with Schizophrenia: 7 (2-19), All Persons: 1 (0-7); Standardized Difference: 0.72  *Number of non-MH visits to PCP (Median (IQR)):*  Individuals without Schizophrenia: 7 (2-16), Individuals with Schizophrenia: 8 (3-21), All Persons: 7 (2-17): Standardized Difference: 0.17 |
| McConnon et al. 2019 [38] | *Outcome: Cancer screening status of people released from provincial correctional facilities and the general population*  Colorectal cancer screening status (overdue on index date): RR 1.53 (1.50-1.55); ARR 1.44 (1.42-1.46) and still overdue at 3 years after index date: RR 1.87 (1.82-1.91); ARR 1.72 (1.68-1.76).  Breast cancer screening status (overdue on index date): RR 2.25 (2.06-2.46); ARR 1.99 (1.83-2.17) and still overdue at 3 years after index date: RR 2.58 (2.29-2.91); ARR 2.29 (2.04-2.58).  *Primary care encounter in 3 years after index date*  Colorectal cancer screening status: ‘All’ (n=3,803): ‘Any primary care encounter in 3 years after index date’ (n=3,292, 86.6%), Median (IQR) 9 (2−20). ‘Still overdue at 3 years after index date’ (n=2,381): ‘Any’ (n=1,946, 81.7%), Median (IQR) 6 (1−17).  Breast cancer screening status: ‘All’ (n=249:) ‘Any primary care encounter in 3 years after index date’(n=226, 90.8%), Median (IQR) 12 (4−24). ‘Still overdue at 3 years after index date’ (n=130): ‘Any’ (n=111, 85.4%). Median (IQR) 7 (2−25) |
| Norris et al. 2021 [39] | *Outcome: Primary care use*  The female incarceration group had high rates of primary care use in all time periods: rate of 19.1 visits per person year  in custody and over 10 visits per person year across periods post release. For the female incarceration group, the rate of primary care use decreased substantially upon release, from 19.1 to 12.2 visits per person year. Female incarceration group, n=6,107.  *In prison:* 19.1 (visits per person year)  *Post-release days* (visits per person year)  0–6: 12.2  7–29: 10.6  30–89: 10.1  90–179: 10.4  180–364: 11.1  365–730: 10.9  Rates of use for this group were significantly higher than rates in the male incarceration group and the female general population group.  Standardized difference for female incarceration groups v male incarceration group.  *In prison:* 0.10  *Post-release days* (visits per person year)  0–6: 0.12  7–29: 0.23  30–89: 0.28  90–179: 0.34  180–364: 0.40  365–730: 0.49.  Standardized difference for female incarceration group. v female general population.  In prison: 0.52  *Post-release days* (visits per person year)  0–6: 0.34  7–29: 0.36  30–89: 0.37  90–179: 0.39  180–364: 0.41  365–730: 0.47 |
| Palis et al. 2022 [40] | Outcome: Mental health services access  Mental health services access (MHSA) was associated with a reduction in the hazard of reincarceration (HR 0.61, 95%CI 0.39-0.94).  For each additional month between release and MHSA, the hazard of reincarceration was increased by 4% (HR 1.04, 95%CI 1.01-1.07). MHSA type, the hazard of reincarceration was significantly higher for emergency outpatient care visits (HR 1.41, 95%CI 1.08-1.83) and other visits (HR 1.41, 95%CI 1.06-1.87) compared with outpatient primary care visits. |
| Palis et al. 2022 [41] | Outcome: Opioid Agonist Treatment (OAT) within 2 days of release.  Approximately 25% (N=3,328) received OAT within 2 days of release.  Stimulant Use Disorder (StUD) diagnosis was associated with OAT (within 2 days of release) in the adjusted analyses (AOR 0.84, 95%CI 0.74–0.95).  Mental ill health and StUD was associated with reduced OAT (AOR 0.73, 95%CI 0.64–0.84) however in the absence of mental ill health this finding was no longer significant [AOR 0.89, 95%CI 0.70–1.13]. People <30 years were less likely to receive OAT (AOR 0.63, 95%CI 0.47–0.86) compared to reference group (>=50 years) |
| Wang et al. 2012 [42] | Outcome: Primary care use  After 12 months of follow-up, 37.7% of Transitions Clinic (TC) participants and 47.1% of Expedited Primary Care (EPC) participants (P=.18) made 2 or more visits to their assigned primary clinic.  The median number of primary care appointments was the same for TC and EPC participants (1 visit; interquartile range [IQR] = 0---5). |
| Wang et al. 2019 [43] | Outcome: Reincarceration  Transitions Clinic (TC) participants were 62% less likely to return to prison for a parole or probation technical violation (AOR 0.38, 95%CI 0.16-0.93) compared with the control group.  TC participants had fewer incarceration days compared with the control group (adjusted incidence rate ratio 0.55, 95%CI 0.35-0.84). After adjustment for incarceration days in people hospitalised, the TC group had significantly less episodes of preventable hospitalisations (IRR 0.46, 95%CI 0.24-0.89) and shorter length of hospital stays (IRR 0.41, 95%CI 0.35-0.84) in comparison to the control group. |
| Young et al. 2015 [44] | Outcome: Primary care physician (PCP) contact  PCP contact prior to the 1-month follow-up interview was 46.5% (n=394).  One-month PCP contact was associated with increased hospital service use (AHR 2.07; 95% CI 1.39 to 3.09), mental health service use (AHR 1.65, 95%CI 1.24-2.19), alcohol and other drug service use (AHR 1.48, 95%CI 1.15-1.90) and subsequent PCP service utilisation (AHR 1.47, 95%CI 1.26-1.72) over 6 months of follow-up.  Further analysis, stratified by repeat offending, demonstrated that PCP contact at 1-month follow-up predicted increased rates of use of hospital (AHR 2.41, 95%CI 1.46-3.99), mental health (AHR 2.15, 95%CI 1.55-2.99), Alcohol and Other Drug service use (AHR 1.48, 95%CI 1.11-1.97), and subsequent PCP services (AHR 1.48, 95%CI 1.25-1.75) for the non-reincarcerated subgroup only. |
